# Chidamide and apatinib are therapeutically synergistic in acute myeloid leukemia stem and progenitor cells

**DOI:** 10.1186/s40164-022-00282-1

**Published:** 2022-05-17

**Authors:** Haijun Zhao, Yuelong Jiang, Fusheng Lin, Mengya Zhong, Jinshui Tan, Yong Zhou, Long Liu, Guowei Li, Manman Deng, Bing Xu

**Affiliations:** 1 Department of Hematology, The First Affiliated Hospital of Xiamen University and Institute of Hematology, School of Medicine, Xiamen University, Xiamen, 361003 People’s Republic of China; 2 Key Laboratory of Xiamen for Diagnosis and Treatment of Hematological Malignancy, No. 55, Shizhen Hai Road, Xiamen, 361003 People’s Republic of China; 3 Department of Hematology, Huizhou Municipal Central Hospital, Huizhou, 516001 People’s Republic of China

**Keywords:** Leukemia stem and progenitor cells, Acute myeloid leukemia (AML), Apatinib, Chidamide, VEGFR, Bcl2, Patient-derived xenografts

## Abstract

**Background:**

Leukemia stem cells (LSCs) are responsible for the initiation and perpetuation of acute myeloid leukemia (AML), and also represent leukemia relapse reservoirs with limited therapeutic approaches. Thus, additional treatment strategies are medical unmet needs to eliminate LSCs.

**Methods:**

Cell counting kit-8 and Annexin-V-FITC/PI assays were used to examine the interaction of chidamide and apatinib on LSC-like cell lines (CD34^+^CD38^−^ KG1α and Kasumi-1 cells) and primary CD34^+^ AML cells. AML patient-derived xenografts were established to investigate the in vivo efficacy of the combined regimen. RNA sequencing, Glutamine uptake assay, oxygen consumption assay, and western blotting were employed to explore the molecule mechanism for the cytotoxicity of chidamide with or without apatinib against LSC-like cell lines and/or primary CD34^+^ AML cells.

**Results:**

In this study, chidamide and apatinib were synergisitc to diminish cell viability and induce apoptosis in CD34^+^CD38^−^ KG1α and Kasumi-1 cells and in CD34^+^ primary AML cells. Importantly, chidamide combined with apatinib had more powerful in reducing leukemia burden and improving prognosis than single drug alone in an AML PDX model without significant adverse effects. Chidamide cytotoxicity was associated with decreasing glutamine uptake. The therapeutic synergy of chidamide and apatinib correlated with reprogramming of energy metabolic pathways. In addition, inactivating the VEGFR function and reducing the anti-apoptotic ability of the Bcl2 family contributed to the synergism of chidamide and apatinib in CD34^+^CD38^−^ KG1α cells and CD34^+^ primary AML cells.

**Conclusion:**

Chidamide in combination with apatinib might be a promising therapeutic strategy to get rid of the population of AML stem and progenitor cells, and thus provide a potentially curative option in the treatment of patients with AML, although further clinical evaluations are required to substantiate the conclusion.

**Supplementary Information:**

The online version contains supplementary material available at 10.1186/s40164-022-00282-1.

## Background

Acute myeloid leukemia (AML) is an aggressively fatal hematopoietic malignancy that originates from leukemia stem cells (LSCs), a rare population with self-renewal capacity and quiescent state [1]. LSCs are resistant to chemotherapies and become leukemia relapse reservoirs responsible for poor prognoses [2, 3]. Thus, elimination of the quiescent LSCs represents a crucial treatment strategy to prevent recurrence and improve the long-term outcome in AML. Recently, the combination of venetoclax and hypomethylating agents elicited favorable clinical outcomes in elderly AML patients and effectively targeted the LSCs population [4–6]. However, recurrent AML still occurs after treatment with these novel combined treatment regimens [7, 8], thereby, posing a major therapeutic challenge, urging pursuit of additional therapeutic interventions to target the LSCs.

Aside from genetic modifications, epigenetic alterations including histone modifications are also involved in the initiation and perpetuation of AML. Histone acetylation is a comprehensively studied posttranslational histone modification that is mainly regulated by two opposing enzymes, histone acetyltransferases (HATs) and histone deacetylases (HDACs) [9]. The HDACs consist of classes I, II, III, and IV that remove the acetyl group from histone lysine residues [10]. Histone deacetylation results in transcriptional inactivation of multiple tumor suppressors [11, 12]. Dysregulation of HDACs plays a critical role in tumorigenesis and is associated with worse clinical outcomes, rendering HDACs as attractive antitumor targets [12]. Indeed, many studies demonstrated that pharmacologically blocking HDACs showed a modest single-agent activity or synergistic antileukemia efficacy in combination with other agents against AML [13–15]. In particular, the HDAC inhibitors were able to effectively eliminate LSC like cells (including AML stem and progenitor cell lines and primary cells) in the preclinical context. Chidamide (CS055) is an orally active HDAC-blocking agent with selectivity against the class I HDAC members, encompassing HDAC 1, 2, 3, and 10. On the basis of its potent antitumor property with acceptable safety profiles, chidamide has been approved for the management of T-cell lymphoma (TCL) in China [16, 17]. Chidamide is capable of enhancing the anti-leukemic effects of chemotherapies in LSC like cells [15, 18], suggesting that it might be a good therapeutic candidate to combine with other antitumor agents to eradicate LSC like cells. The use of chemotherapeutic drugs in AML is often restricted by their significant adverse effects, therefore requiring the development of alternative combination therapies with chidamide to eliminate LSC like cells with minimal toxicities.

Apatinib is an oral VEGFR2 tyrosine kinase inhibitor derived from valatinib and has shown impressive antitumor responses in solid tumors in numerous clinical trials [19–21]. Preclinical data have revealed that apatinib had the ability to suppress cell proliferation and induce apoptosis of AML cells without impact on normal hematopoietic cells. Interestingly, previous studies found that LSC-like cells seemingly had a similar sensitivity to the cytotoxic effect of apatinib when compared with non-LSC-like cells [22, 23]. Given the fact that both chidamide and apatinib are cytotoxic against the LSC-like cells, we aimed to investigate whether the two drugs produce synergistic antileukemic effects on the LSC-like models.

## Materials and methods

### Reagents

Chidamide (CS055, purity of > 95%) was purchased from Chipscreen Bioscience Co., Ltd. (Shenzhen, Guangdong, China) and dissolved in dimethyl sulfoxide (DMSO) (Invitrogen Corp., Waltham, MA, USA) to obtain a stock solution of 50 mM. Apatinib (YN968D1) was kindly donated by the Jiangsu Hengrui Medicine Company (Lianyungang, Jiangsu, China) and dissolved in DMSO (Sigma-Aldrich Corp., St. Louis, MO, USA) to obtain a 100 mM stock solution for in vitro experiments. Subsequently, it was diluted to the designated concentrations with the culture media for the later experiments. For in vivo administration, the compounds were suspended in a 0.2% (w/v) carboxymethyl cellulose-sodium (CMC-Na) suspension for the oral gavage.

### Cell lines and cell culture

Both KG1α and kasumi1 cell lines were used as LSC-like cell models, as they were CD34 positive and CD38 negative in over 99% of cells (Additional file 1: Fig. S1). KG1α cells were cultured in Iscove’s modified Dulbecco’s medium (IMDM, Gibco BRL, Rockville, MD, USA) with 100 U/mL penicillin and 100 μg/mL streptomycin (1 × P/S) and 10% fetal bovine serum (FBS) (Natocor Biologicals, Cordoba, Argentina) at 37 °C in a 100% humid atmosphere with 5% CO_2_. The Kasumi-1 cells were cultured in the RPMI-1640 (HyClone™, Logan, UT, USA) with 1 × P/S and 20% FBS. The CD38 microbeads (Miltenyi Biotech, Bergisch Gladbach, Germany) were used to isolate CD38^−^ cells, depleting the CD38^+^ cells.

The CD34^+^ primary AML cells were sorted from the bone marrow of the AML patients (n = 20) using magnetic cell sorting and the normal hematopoietic stem cells (n = 8) were obtained from the healthy donors for the hematopoietic stem cell transplantation. The study protocol was approved by the First Affiliated Hospital of Xiamen University Ethics Review Board following the Declaration of Helsinki. Acquisition of bone marrow samples was performed with the informed consent of the patients. The clinical characteristics of the AML patients are summarized in Table 1. The mononuclear cells were isolated by density gradient centrifugation using Lymphoprep (BD, Franklin Lakes, NJ, USA) and cultured in the IMDM (HyClone, Thermo Fisher Scientific, Eugene, OR, USA) supplemented with 10% FBS (Gibco, Life Technologies, Eugene, OR, USA), 100 U/mL penicillin and 100 μg/mL streptomycin (1 × P/S) at 37 °C. The CD34 microbeads (Miltenyi Biotech) were used to enrich the CD34^+^ cells according to the manufacturers’ recommendations.

**Table 1:** The IC50 values of KG1a and Kasumi cells treated with Chidamide alone or in combination with Apatinib

Drugs	IC50 (uM) of KG1α		Fold	*P*	IC50 (uM) of Kasumi-1		Fold	*P*
	Single	Combination			Single	Combination		
Chidamide	8.05 ± 2.13				8.37 ± 3.02			
Apatinib (2.5 uM) + Chidamide		6.04 ± 1.45	1.33	< 0.01		6.44 ± 1.76	1.30	< 0.01
Apatinib (5.0 uM) + Chidamide		3.58 ± 1.62	2.25	< 0.01		4.10 ± 1.35	2.04	< 0.01
Apatinib (10.0 uM) + Chidamide		1.99 ± 0.87	4.05	< 0.01		2.03 ± 0.73	4.12	< 0.01

### Cell proliferation assay

The cytotoxic effects of chidamide with or without apatinib on CD34^+^CD38^−^KG1α and CD34^+^CD38^−^Kasumi-1 cells were determined using cell counting kit-8 (CCK-8, Dojindo Laboratories, Kumamoto, Japan) assay. The cells (2 × 10^4^ cells/well) were seeded in 96-well plates containing 100 μL growth medium, treated with the designated doses of chidamide or apatinib alone or in combination, and incubated at 37 °C in a 5% CO_2_ incubator with 100% humidity for 48 h. The CCK-8 reagents (10 μL/well) were then added and incubated for an additional 2 h. Finally, the absorbance was detected at 450 nm wavelength in a microplate reader (ELx800, BioTek Laboratories, Shoreline, WA, USA). The numerical values from three independent triplicates were expressed as a percentage of dead cells compared to the control from the same experiment. Statistical analyses were performed using SPSS 20.0 and the IC50 values were determined.

### Annexin V-FITC/PI double-staining apoptosis assay

To assess the apoptosis, the cells were treated with chidamide or apatinib alone or in combination for 48 h and stained with the Annexin-V-FITC/PI (eBioscience, San Diego, CA, USA) for 15 min at room temperature in the dark according to the manufacturer’s instructions. The cells were then analyzed by NovoCyte flow cytometer and the data were analyzed using the NovoExpress software (ACEA Biosciences, Inc., San Diego, CA, USA) to determine the percentage of the Annexin-V positive (apoptotic) cells.

### RNA sequencing

KG1α cells were treated with apatinib (5 µM) and chidamide (5 µM) alone or in combination for 24 h. Subsequently, total RNA samples of KG1α cells treated as described above was prepared and then referred to the RNA sequencing (RNA-seq) using the illumine Hiseq 2500. Briefly, the prepared RNA samples were extracted and fragmented into short fragments which was used as templates for the double-stranded cDNA synthesis. The synthesized cDNA was purified and enriched by polymerase chain reaction (PCR) amplification, after which the library products were sequenced.

### Glutamine uptake assay

A total of 5 × 10^4^ cells/well were cultured in 96-well plates containing advanced DMEM (Thermo Fisher Scientific) with 10% fetal bovine serum (FBS, Thermo Fisher Scientific) and 2 mM glutamine, and then transferred to a CO_2_ incubator set at 37 °C, 100% humidity, and 5% CO2 at specified times. The supernatant media were collected to measure the remaining glutamine using a glutamine assay kit (Abcam, Cambridge, MA, USA) following the manufacturer's instructions.

### Oxygen consumption assay

Following the manufacturer's manual, the cellular oxygen consumption rate (OCR) was tested using a Seahorse XF Extracellular Flux Analyzer (Seahorse Bioscience, MA, USA). In brief, KG1α cells (3 × 10^5^ cells per well) were exposed to 5 µM chidamide and 5 µM apatinib alone or in combination for 24 h, and then resuspended in XF media and plated into a XFe-96 plate, followed by real-time measurement of OCR in the XFe-96 Extracellular Flux Analyzer. The OCR was measured in XF medium (non-buffered DMEM medium containing 10 mM glucose and 1 mM sodium pyruvate) under basal conditions, as well as responded to 1 μM of oligomycin, 1 μM of FCCP (carbonylcyanide-4-(trifluoromethoxy)-phenylhydrazone) and 1 μM of antimycin and rotenone (Sigma-Aldrich, MO, USA), respectively.

### Western blotting analysis

The whole-cell lysates (50 μg/lane) from each sample were subjected to 8% or 10% SDS-PAGE and then the proteins were transferred to the PVDF membranes (Millipore Corp., Burlington, MA, USA). After blocking the nonspecific binding for 1 h in TBS-T with 5% nonfat milk, the membranes were incubated with the primary antibodies (VEGFR, rabbit monoclonal, 1:1000; p-VEGFR, rabbit monoclonal, 1:1000; β-actin, rabbit monoclonal, 1:1000; Cell Signaling Technology, Inc., Danvers, MA, USA) overnight at 4 °C and then incubated with a secondary HRP-conjugated monoclonal antibody (1:10,000, Cell Signaling Technology Inc.) for 1 h at room temperature. The expression of target proteins was detected using an enhanced chemiluminescence western blotting detection kit (Santa Cruz Biotech., Santa Cruz, CA, USA) following the manufacturer's instructions.

### Patient-derived xenograft models of the AML

The primary cells from the AML patients were collected from the bone marrow aspirates following the approved protocols. To establish the patient-derived xenograft (PDX) model, the leukemic blast cells were injected intravenously into the NSG mice and monitored for disease progression, as demonstrated by the presence of human CD45 + (hCD45 +) cells in the peripheral blood. The analysis of the extent of the hCD45 + cell infiltration in the femur and tibia bones extracted from two engrafted animals was used to confirm that they harbored a heavy tumor burden before the start of the therapy. To determine the drug efficacies, the mice were administered by oral gavage with vehicle (methylcellulose/tween− 80), apatinib (20 mg/kg twice a week), or chidamide (15 mg/kg daily) for 5 days. To determine the drug response in the mice in all experimental groups, we measured the percentage of hCD45 + AML cell infiltration in the bone marrow leukocytes and the absolute counts of the hCD45 + cells per femur and tibia in the PDX models from each experimental group.

### Statistics

All data analyzed in this study were presented as mean ± standard deviation (SD) from three independent experiments. All statistical analyses were performed using GraphPad Prism 8.3.0 software. Variables between two groups were compared using the two-tailed Student’s t test. Comparisons among multiple groups were performed using the One-way analysis of variance (ANOVA) followed by the Bonferroni post hoc test. P < 0.05 was considered as statistically significant. P < 0.05 was considered statistically significant.

## Results

### Chidamide and apatinib are synergistic to eliminate leukemia stem-like cells

**Fig. 1:**
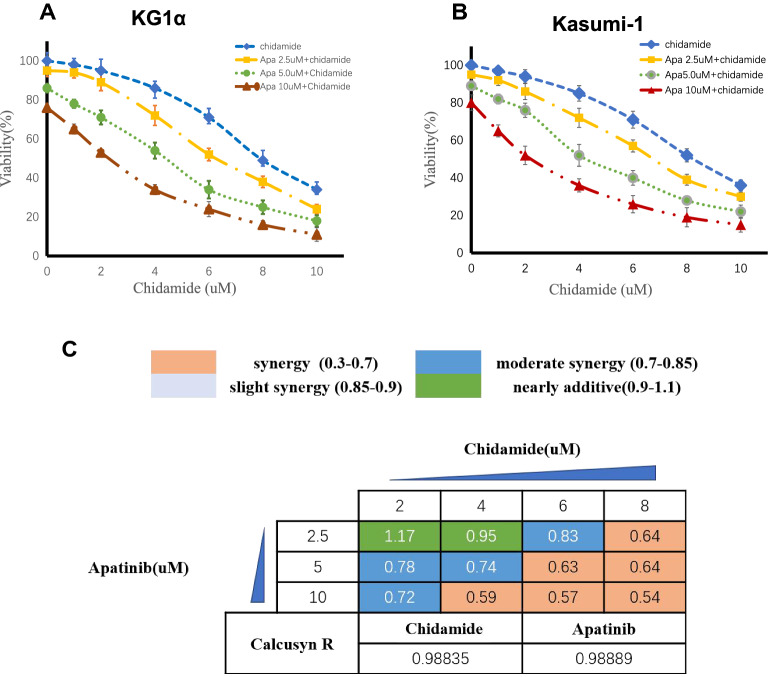
Chidamide and apatinib are synergistic to decrease the cell viability of CD34^+^CD38^−^ KG1α and Kasumi-1 cells. The CCK8 assay kit was used to determine the cell viability of both CD34^+^CD38^−^ KG1α **(A)** and Kasumi-1 cells **(B)** treated with the designated doses of apatinib (apa) and chidamide alone or in combinations for 48 h. **C** The combination index (CI) was calculated with the Chou-Talalay method based on the results described in **A**. The CI values of < 1, = 1, or > 1 indicate synergistic, additive, or antagonistic activity, respectively

Our previous work has demonstrated that chidamide could act as a sensitizer of idarubicin, daunorubicin, and cytarabine, three conventional cytotoxic drugs, against LSC-like cells [18]. Apatinib was also proven to be a killer of LSC-like cells in vitro [22]. In the study, we sought to investigate the treatment interaction of chidamide in combination with apatinib in LSC-like cells. CD34^+^CD38^−^ KG1α and Kasumi-1 cells were here used as the typical cellular models of LSC-like cell lines based on previous published series. Consequently, chidamide treatment resulted in a dose-dependent cell viability reduction in CD34^+^CD38^−^ KG1α and Kasumi-1 cells (Fig. 1A, B), with the IC50 values of 8.05 μM and 8.37 μM, respectively (Table 1). The addition of apatinib to chidamide significantly enhanced the anti-LSC activity of chidamide (Fig. 1A, B) and greatly lowered the IC50 values of chidamide in CD34^+^CD38^−^ KG1α and Kasumi-1 cells (Table 1). Next, the Chou-Talalay method was employed to calculate the combination index (CI) values on the basis of cell viability results, where the CI values of < 1, = 1, or > 1 indicate synergistic, additive, or antagonistic activity, respectively. As shown in Fig. 1C, all dose combinations of chidamide and apatinib except the lowest dose combination were < 1, suggesting that the two drugs act synergistically each other to eliminate the LSC-like cell lines.

**Fig. 2:**
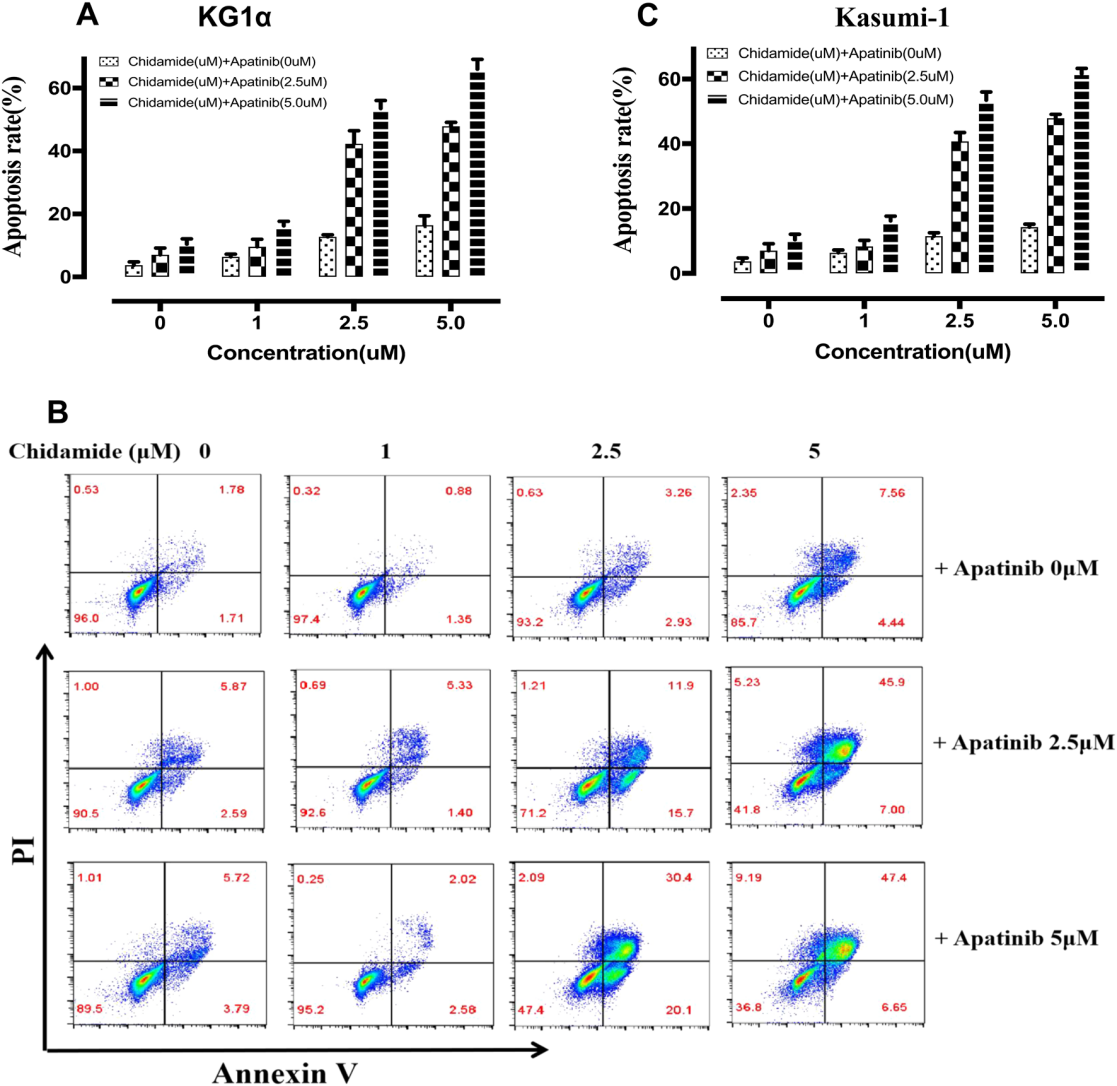
The therapeutic cooperation of chidamide coupled with apatinib in promoting apoptosis in CD34^+^CD38^−^ KG1α and Kasumi-1 cells. The Annexin V/PI dual staining followed by flow cytometry detection was employed to analyze the apoptotic percentage of CD34^+^CD38^−^ KG1α **(A)** and Kasumi-1 **(B)** cells exposed to apatinib and chidamide alone or in combinations for 48 h. **C** The representative flow plots of the cell apoptosis obtained from **A**, **B**

The Annexin V/PI staining assay was carried out to confirm the synergistically LSCs-killing effect of chidamide combined with apatinib in CD34^+^CD38^−^ KG1α and Kasumi-1 cells. Either apatinib or chidamide alone caused a slight to moderate cell apoptosis, while the combination of the two drugs led to significant cell apoptosis in a dose-dependent manner (Fig. 2A–C). Taken together, these findings indicate that chidamide synergizes with apatinib to decrease the cell viability and increase the apoptosis of LSC-like cells.

### Chidamide and apatinib act cooperatively against the primary CD34^+^ AML cells but spare the normal hematopoietic cells

**Fig. 3:**
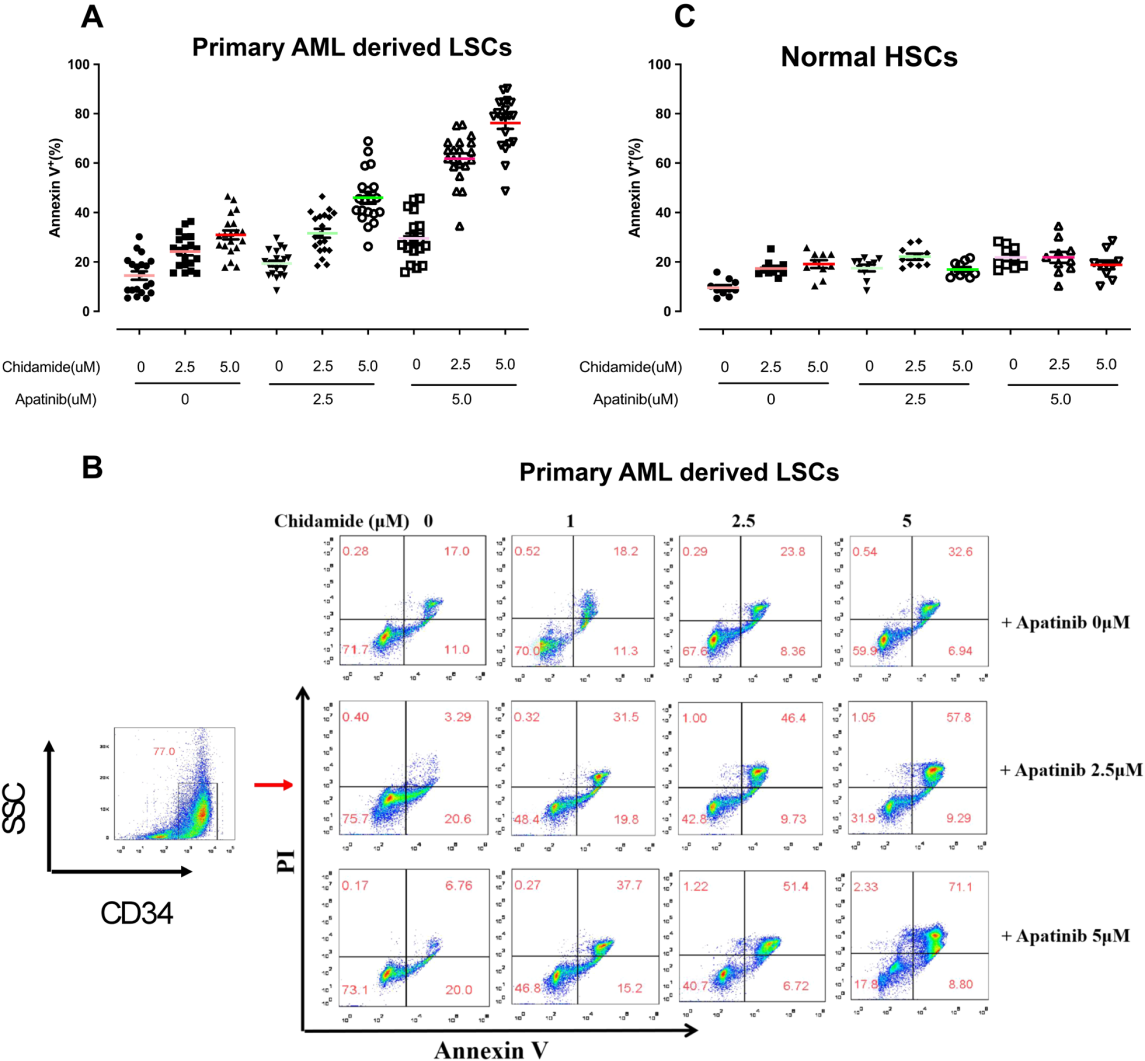
Chidamide and apatinib act synergistically against the primary CD34^+^ AML cells but spare the normal hematopoietic cells. Twenty primary AML bone marrow samples and 8 normal hematopoietic cells were collected and centrifuged using Lymphoprep. These primary AML cells and hematopoietic cells were separately treated with apatinib and chidamide alone or in combination for 24 h. At the end of the treatment, cells were then sorted using the human CD34 antibody and stained with Annexin V/PI kit followed by flow cytometry analysis. **A, C** The apoptosis-inducing ability of apatinib and chidamide alone or in combinations in primary CD34^+^ AML cells and normal HSCs. **B** The representative flow plot of cell apoptosis in the primary CD34^+^ AML cells treated as described in **A**

The antileukemic effect of chidamide and apatinib alone or in combination was analyzed in primary CD34 positive (CD34^+^) AML cells and normal hematopoietic cells. In total, 20 primary AML bone-marrow samples were collected and used for the isolation of primary CD34^+^ leukemia blasts, where CD34^+^ is a well-characterized biomarker of leukeima stem and progenitor cells. The clinical characteristics of the 20 AML patients are summarized in Table 2. Consistent with the results observed in the LSC-like cell lines, cotreatment of primary CD34^+^ AML cells with chidamide and apatinib resulted in significant increases in the percentage of the apoptotic cells when compared with each single agent (Fig. 3A, B). Normal hematopoietic cells were collected and enriched to assess whether the drug comibation could remarkably affect the normal hematopoietic processess. A total of 10 hematopoietic donors derived bone marrow mononuclear cells (BMMCs) were extracted and treated with chidamide alone or combined with apatinib for 24 h. In marked contrast, chidamide and apatinib alone or in combination had marginal apoptosis-inducing activity in the normal BMMCs, indicating that the combinational regimen might be safe for patients with AML in future clinical studies (Fig. 3C). Together, these results suggest that the combination of chidamide and apatinib might preferably target the AML blasts while spare the normal hematopoietic cells.

**Table 2:** The clinical characteristics of 20 cases diagnosed with de novo or R/R AML

Patient No	Age (yrs)	Gender	WBC (× 10^9^)	LDH (U/L)	Blasts (%)	Karyotype	Molecular features
1	68	F	4.85	Normal	24	Normal	/
2	66	M	48.34	High	36	46, XY, t (2;22)	TET2
3	55	F	21.38	High	69	Complex	NPM1, TET2, ASXL1
4	57	M	47.34	High	74	46, XY, t (8;21)	AML-ETO
5	32	M	324.4	High	92	46, XX, inv (16)	CBFB-MYH11
6	60	M	52.12	Normal	43	Normal	CEBPA, TET2
7	64	F	16.12	High	62	Normal	IDH, Runx1
8	28	F	15.63	High	52	46, XY, inv (16)	CBFB-MYH11, ASXL1, CEBPA
9	44	M	23.56	Normal	46	Normal	/
10	9	F	581.8	High	92	ND	/
11	11	M	48.65	High	57	Normal	/
12	6	F	231.2	High	78	Complex	FLT3-ITD
13	5	M	180.3	High	43	46, XX, del (14) (q32)	/
14	10	F	53.1	High	64	Normal	c-KIT
15	58	M	45.3	Normal	36	45, XY, − 7	TP53
16	46	F	153.2	High	76	Complex	FLT3-ITD
17	32	F	201.2	High	91	46, XX	ASXL1
18	40	M	53.4	High	45	45, XY, − 5	/
19	19	M	75.3	High	58	46, XY	DNMT3A
20	23	F	121.5	Normal	48	Normal	CEBPA

### Chidamide and apatinib are active in an AML patient-derived xenograft (PDX) mouse model

**Fig. 4:**
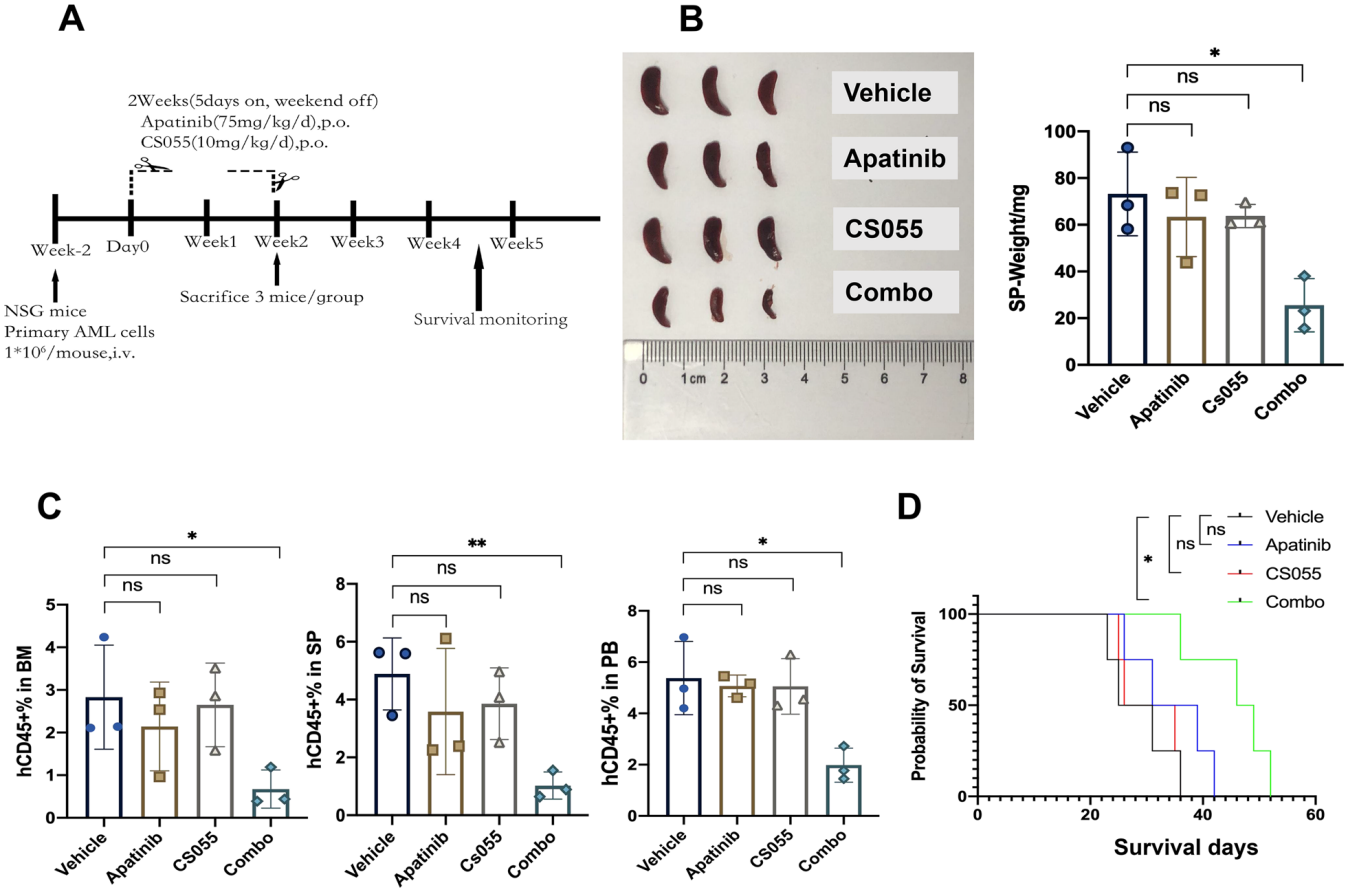
Chidamide synergizes with apatinib to abrogate the leukemia burden and prolong the survival in an AML PDX model. **A** The schematic plan of the in vivo experiment of the AML PDX model. **B** The spleen image (left panel) and weight (right panel) from the PDX mice treated with distinct groups. **C** The analysis of human CD45 (hCD45) percentages in bone marrow (BM, left panel), spleen (SP, middle panel), and peripheral blood (PB, right panel) in the four different treatment groups. **D** Kaplan Meier curve of the primary AML PDX mice administered with Vehicle, apatinib, chidamide and the combined regimen at the planned treatment timepoints. CS055 and Combo mean chidamide and the combination of chidamide and apatinib

To assess the antileukemic efficacy of the combined treatment in vivo, an AML PDX model was established by the intravenous inoculation of the AML cells (patient #16) with complex karyotype and FLT3-ITD mutation. Human CD45 (hCD45) staining was used as a leukemia burden biomarker and its proportion was monitored as planned. These AML-bearing mice were randomized into four distinct treatment groups when the hCD45 percent was of ≥ 1%, and they were subjected to a consecutive two-week treatment plan with 5 days on and 2 days off (Fig. 4A). At the end of the treatment plan, three mice of each group were euthanized to measure leukemia burden, while the remaining mice were kept to analyze the survival curve. Either apatinib or chidamide administration alone only resulted in a slight reduction in spleen size and weight, whereas the combined regimen dramatically ameliorated the disease-associated splenomegaly (Fig. 4B). The FACS analysis revealed that cotreatment with chidamide and apatinib remarkably attenuated the tumor burden in the murine bone marrow (BM), spleen (SP), and peripheral blood (PB) (Fig. 4C), reflected by fewer hCD45 positive cells in the combined treatment group than in the single drug groups. Importantly, the coadministration of chidamide and apatinib substantially prolonged the survival period of the AML PDX models (Fig. 4D). Collectively, the combined treatment of chidamide and apatinib is potent to reduce leukemia burden and extend the survival in AML in vivo.

### Dysregulation of metabolic pathways contributes to the cytotoxicity of chidamide against LSC-like cells

**Fig. 5:**
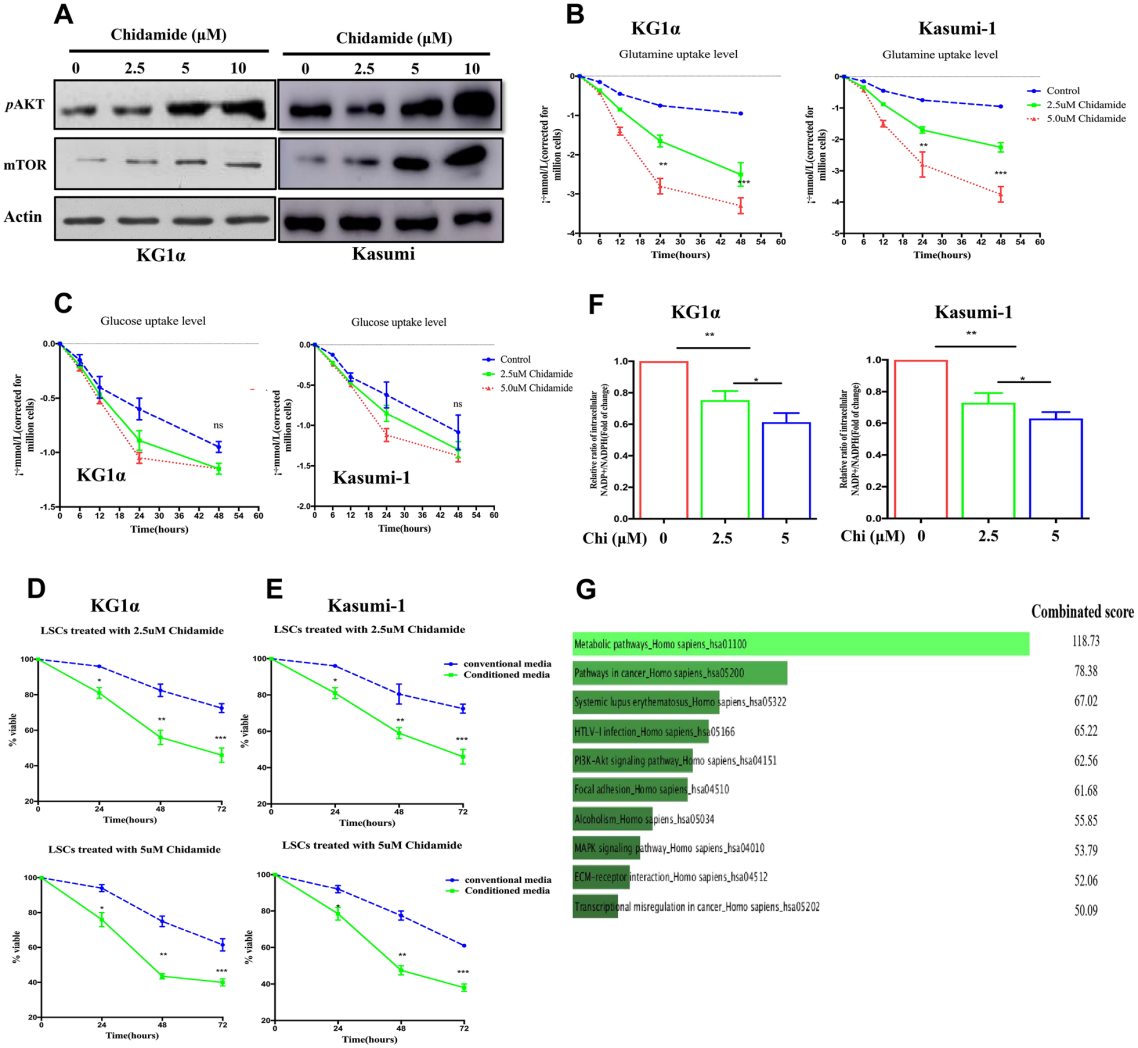
Dysregulation of metabolic pathways contributes to the cytotoxicity of chidamide against LSC-like cells. **A** Immunobloting analysis of the levels of mTOR and the phosphorylation of AKT (p-AKT) in chidamide-treated CD34^+^CD38^−^ KG1α and kasumi-1 cells. Assessment of the uptake levels of glutamine **(B)** and glucose **(C)** in CD34^+^CD38^−^ KG1α and kasumi cells treated with designated concentrations of chidamide. **D** Analysis of the relative NADP + /NADPH ratio in CD34^+^CD38^−^ KG1α and kasumi-1 cells exposed to chidamide. **E, F** Quantification of the cell viability of CD34^+^CD38^−^ KG1α and kasumi-1 cells treated as described in Fig. 5B in the presence of the culture medium with or without glutamine deprivation. **G** The top 10 altered signaling pathways affected by the combination of apatinib and chidamide in the RNA sequencing assasy

The AKT/mTOR pathway plays diverse roles in tumor initiation and maintenance, including involvement in the modulation of metabolic processess. Our results observed that chidamide-treated CD34^+^CD38^−^ KG1α and kasumi cells showed dose-dependent upregulation of AKT and its downstream target mTOR (Fig. 5A). This observation prompted us to investigate whether chidamide exposure could affect the glucose and glutamine metabolisms in LSC-like cells. Consequently, chidamide treatment resulted in significant enhancement of glutamine uptake, manifested by significant reduction of the glutamine levels in the culture medium in CD34^+^CD38^−^ KG1α and kasumi cells (Fig. 5B). However, glucose uptake did not affect by chidamide exposure in both cell lines (Fig. 5C). Furthermore, we demonstrated that the glutamine deprivation obviously enhanced the antileukemic activity of chidamide in LSC-like cells in a dose-dependent manner (Fig. 5D, E). In addition, chidamide induced a decreased ratio of NADP + /NADPH in the mitochondrial matrix (Fig. 5F), which was required for the ROS quenching. These findings unravel that the energy metabolism is reprogrammed by chidamide treatment in LSC-like cells.

**Fig. 6:**
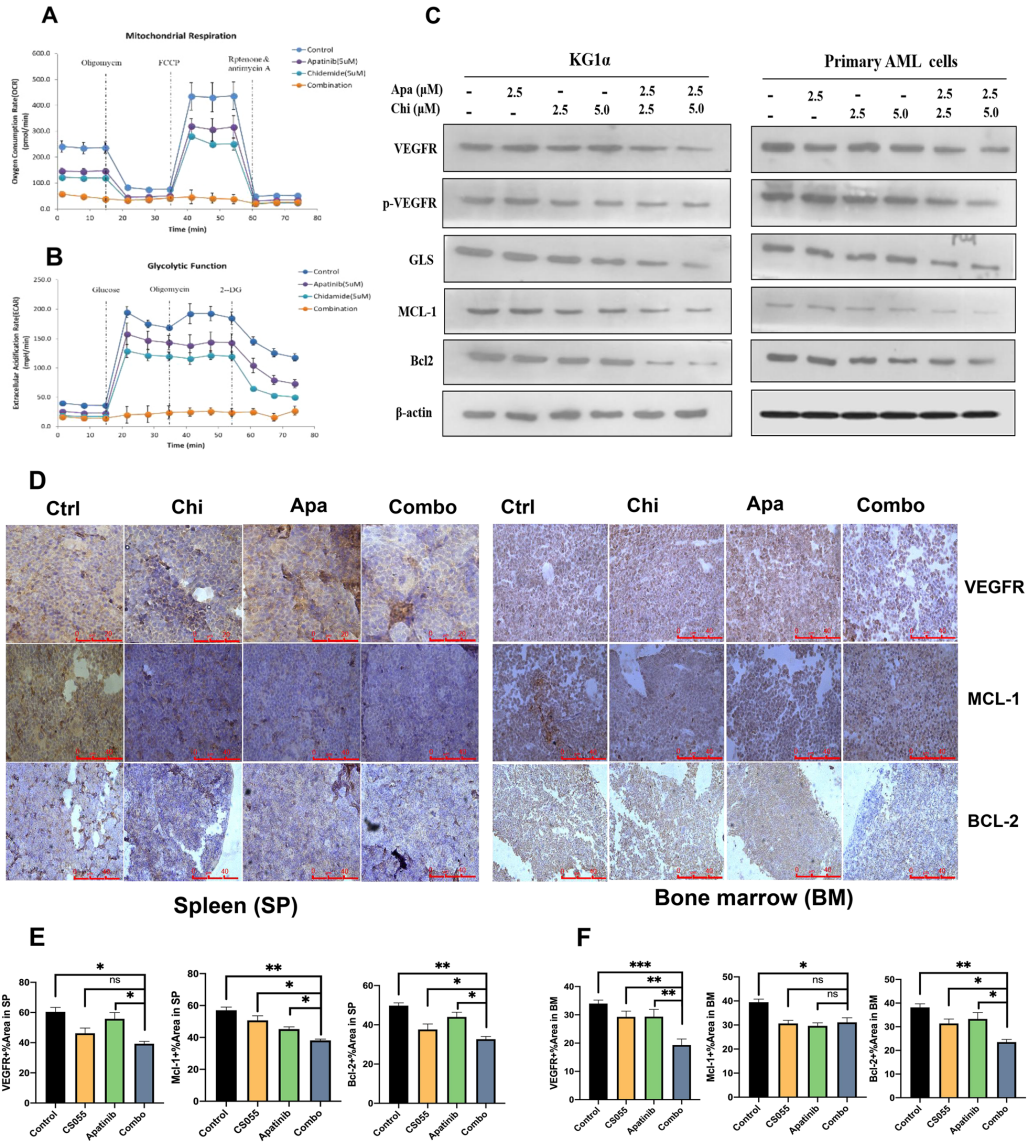
Chidamide plus apatinib impact the glucose metabolism independent of the mitochondrial activity and perturb the VEGFR signaling. **A, B** Examination of the mitochondrial respiration and glycolytic function in CD34^+^CD38^−^ KG1α cells treated with chidamide and apatinib alone or in combination. **C** Western blotting analysis of the expression levels of the indicated proteins in CD34^+^CD38^−^ KG1α and primary AML cells exposed to designated doses of apatinib and chidamide alone or in combination. Apa and Chi represent apatinib and chidamide, respectively. **D** Immunohistochemistry analysis of the exprssion levels of VEGFR, BCL2 and MCL1 in both spleen (left panel) and bone marrow (right panel) in AML PDX mice administered with vehicle, chidamide, apatinib, or the combined treatment groups. **E, F** Quantification of the protein levels of VEGFR, BCL2 and MCL1 in spleen (**E**) and bone marrow (**F**) in AML PDX mice administered with distinct treatment groups. Apa, Chi and Combo represent apatinib, chidamide and the combination of the two drugs, respectively. *, ** and *** indicates *p* < 0.05, *p* < 0.01, *p* < 0.001, respectively. ns indicates not significant

In the study, RNA sequencing was employed to elucidate the underlying mechanism of action for the synergistic activity of chidamide and apatinib in LSC-like cells. The sequencing results identified many significantly altered signaling cascades in CD34^+^CD38^−^KG1α treated with the combined regimen. Figure 5G listed the top 10 dysregulated signaling pathways with metabolic alteration being the top one (Table 3). In contrast to chidamide and apatinib alone, the combined treatment induced a significant decrease in the mitochondrial oxidative metabolism (Fig. 6A) and glycolysis (Fig. 6B). These results indicates that the perturbation of glucose metabolism at least in part contributes to the synergy of chidamide and apatinib in LSC-like cells.

**Table 3:** Genome-wide gene expression profiles of KG1a cells treated with apatinib and chidamide alone or in combination

Gene	Metabolic function	Mean fold change (log2)	Mean FDR
MPI	Carbohydrates metabolism	− 3.13	1.48E−18
MECR	Fatty acids synthesis	− 2.24	6.50E−12
PLCH1	Phosphatidylinositol metabolism	− 2.24	1.85E−05
PIGK	Glycosylphospatidylinositol biosynthesis	− 2.23	3.30E−04
IDH3B	TCA cycle enzyme	− 2.09	4.79E−05
GLS	Glutamine metabolism	− 2.07	2.70E−04
NDUFB3	Electon transport chain subunit	− 2.06	3.53E−09
GAMT	Creatine biosynthesis/Fatty acid oxidation	− 2.03	3.78E−06
ST6GALNAC1	Protein glycosylation	− 1.88	1.83E−05
NDUFAB1	Electron transport chain subunit	− 1.84	2.70E−03

Additionally, we noticed that the combined regimen dramatically downregulated the levels of VEGFR and its phosphorylated form (p-VEGFR) in CD34^+^CD38^−^KG1α and primary CD34^+^ AML cells (Fig. 6C). Two important antiapoptotic proteins, MCL1 and BCL2, were also synergistically decreased by the combination of the two drugs (Fig. 6C). The glutaminase (GLS), a critical enzyme responsible for the conversion of glutamine to glutamate, plays an essential role in the tumor cell metabolism, growth, and proliferation. Importantly, we confirmed that chidamide in combination with apatinib significantly downregulated the protein levels of VEGFR, BCL2 and MCL1 in spleen and bone marrow tissues from AML PDX xenografts (Fig. 6D, E). In this study, we observed that chidamide coupled with apatinib were able to cooperatively downregulate the levels of GLS in LSC-like cells.

## Discussion

The effective targeting of leukemia stem cells (LSCs), a specific subset responsible for leukemogenesis and tumor recurrence, has the potential to improve the clinical outcomes and even to cure AML. Nevertheless, there are limited therapeutic interventions to eliminate the LSCs. In this study, we provided a novel treatment strategy by combining chidamide with apatinib to eliminate the leukemia stem and progenitor cell population in AML. The combination of chidamide and apatinib was synergistic to reduce the cell viability and to promote apoptosis in LSC-like cell ines (including CD34^+^CD38^−^ KG1α and kasumi cells) and AML progenitor cells (primary CD34^+^ AML cells). More notably, chidamide synergized with apatinib to revoke the tumor burden and prolong the survival in an AML derived PDX model. In addition, this combination regimen appeared to be well tolerated as it had marginal cytotoxic effects on normal hematopoietic cells ex vivo and did not impact the bodyweight of the PDX mice as well as showed no other therapeutic-related toxicities in vivo.

The pieces of evidence have showed that the HDAC inhibitors (HDACi) are active in diverse malignant disorders including AML, while monotherapy with the HDACi leads to about 30% of clinical responses. This necessitates searching for combinations with other antitumor drugs to improve HDACi antitumor efficacy. Chidamide, a novel oral HDACi, coupled with other chemotherapeutics or targeted agents, was effective to target leukemia stem and progenitor cells [15, 18]. In agreement with previous reports, the present study observed that LSC-like cell lines and primary CD34^+^ AML progenitor cells were moderately susceptible to the cytotoxicity of chidamide. Recent studies found that HDACs might have important roles outside of transcriptional modulation, such as impacting the metabolic process [24, 25]. Moreover, the pharmacological blocking of HDAC activity impacts various metabolic processes [26, 27]. In the current study, we demonstrated that chidamide-treated LSC-like cell lines had increased glutamine uptake but did not affect glucose uptake. Deprivation of the glutamine from the culture medium made the LSC-like cells more sensitive to chidamide treatment. These results suggested that glutamine metabolism was involved in the moderate anti-LSCs ability of chidamide, providing a rationale to potentiate elimination of LSCs by the combined treatment of chidamide with other compounds targeting glutamine metabolism. In addition, the ratio of NADP + /NADPH was significantly decreased by the chidamide treatment.

Metabolic reprogramming is a hallmark of malignant diseases and plays essential roles in tumor initiation, progression, and recurrence by supplying sufficient nutrients and metabolites to meet the high demands of tumor cells [28–30]. This function provides an opportunity for the development of antitumorigenic compounds to perturb the tumor metabolic reprogramming. In this study, the RNA sequencing results revealed that apatinib along with chidamide strikingly influenced several signaling pathways. Among these pathways, alteration of metabolic pathways was one of the most principal one, suggesting that disruption of metabolism was closely associated with the synergy of chidamide and apatinib in eliminating LSC-like cells. Unlike the solid cancer stem cells, multiple studies have proven that LSCs are mainly dependent on the oxidative phosphorylation (OXPHOS) with a lower glycolytic reserve [31]. Thus, therapies targeting suppression of OXPHOS have showed significant antitumoral efficacy on the LSC subset [32, 33]. In the current study, the combination of apatinib and chidamide resulted in significant reduction of mitochondrial respiration compared to each single drug alone. This finding implied that blocking the LSC-specific dependency on OXPHOS at least in part contributed to the therapeutic cooperation of the two drugs. Additionally, the activity of glycolysis in LSCs was also repressed by the drug combination.

The vascular endothelial growth factor (VEGF) and its receptors (VEGFRs) are important proangiogenic mediators and are deregulated in tumorigenesis [34, 35]. There are three different VEGFRs, comprising of VEGFR1 (Flt1), VEGFR2 (KDR), and VEGFR3 (Flt4). The mitogenic and angiogenic-promoting effects of VEGF are primarily mediated through VEGFR2 [36, 37]. Blocking the role of the VEGF/VEGFR signaling is cytotoxic against multiple neoplasms, including AML [22, 38, 39]. Our previous study revealed that a novel small molecule VEGFR2 antagonist was potent to kill AML cells, including LSC-like cells. The mechanism of action for the anti-leukemia effect of apatinib on AML was associated with downregulating the level of VEGFR phosphorylation (p-VEGFR) and its downstream pro-survival pathways [22]. In the present study, the p-VEGFR level was obviously lower in the combined treatment group than in the single drug alone group, suggesting that inactivation of the VEGFR pathway at least partially contributed to the synergistic effects of apatinib and chidamide on LSC-like cells.

The Bcl2 family has long been identified for its critical role in the control of cell apoptosis [40]. This family can be classified into three distinct subfamilies: antiapoptotic, BH3-only (proapoptotic), and pore-forming or ‘executioner’ (proapoptotic) families. Both MCL1 and Bcl2 are antiapoptotic proteins [41]. Dysregulation of MCL1 and Bcl2 is noted in cancers and correlates with worse clinical outcomes [42, 43], thereby rendering them excellent treatment targets. Not surprisingly, pharmacological MCL1 or Bcl2 inhibition displays robust antitumoral effects on tumors, with a special success on AML [6, 27, 44–46]. In this study, we observed that either apatinib or chidamide could diminish the levels of MCL1 and Bcl2. Of great importance, apatinib and chidamide were therapeutically synergistic in downregulating the levels of MCL1 and Bcl2 proteins. These findings uncovered that the promotion of cell apoptosis through suppressing the function of the antiapoptotic proteins was involved in the therapeutic synergy of the two drugs in LSC-like cells.

The study provided attractive preclinical evidence of the combined treatment with chidamide and apatinib to eliminate leukemia stem and progenitor cells in AML models. The synergism of chidamide combined with apatinib was mechanistically associated with the reprogramming of energy metabolic pathways, including attenuation of the aerobic and anaerobic metabolic processes. Weakening the VEGFR-mediated proangiogenic activity and blocking the anti-apoptotic ability of the BCL2 family were also implicated in the therapeutic cooperation of chidamide and apatinib against leukemia stem and progenitor cells. Overall, chidamide plus apatinib might become a promising therapeutic avenue to eradicate leukemia stem and progenitor cells and thus offer a potential curative option in the treatment of patients with AML, although the conclusion requires further clinical investigation.

## Supplementary Information

**Additional file 1: Supplementary Figure 1.** The expression of CD34 and CD38 in KG1а and Kasumi-1 cells.

## Data Availability

The datasets presented in this study are available from the corresponding authors with reasonable requests.

## Abbreviations

LSC: Leukemia stem cell
AML: Acute myeloid leukemia
HATs: Histone acetyltransferases
HDACs: Histone deacetylases
HDACi: Histone deacetylases inhibitors
TCL: T-cell lymphoma
OXPHOS: Oxidative phosphorylation
PDX: Patient derived xenograft
BMMCs: Bone marrow mononuclear cells
GLS: Glutaminase
VEGF: Vascular endothelial growth factor
VEGFRs: Vascular endothelial growth factor receptors
CCK-8: Cell counting kit-8
PCR: Polymerase chain reaction
CI: Combination index
