# Supplementary figures and images for: Chidamide and apatinib are therapeutically synergistic in acute myeloid leukemia stem and progenitor cells
Source: Exp Hematol Oncol. 2022 May 17;11:29. doi: 10.1186/s40164-022-00282-1 (PMC9112613; doi:10.1186/s40164-022-00282-1)

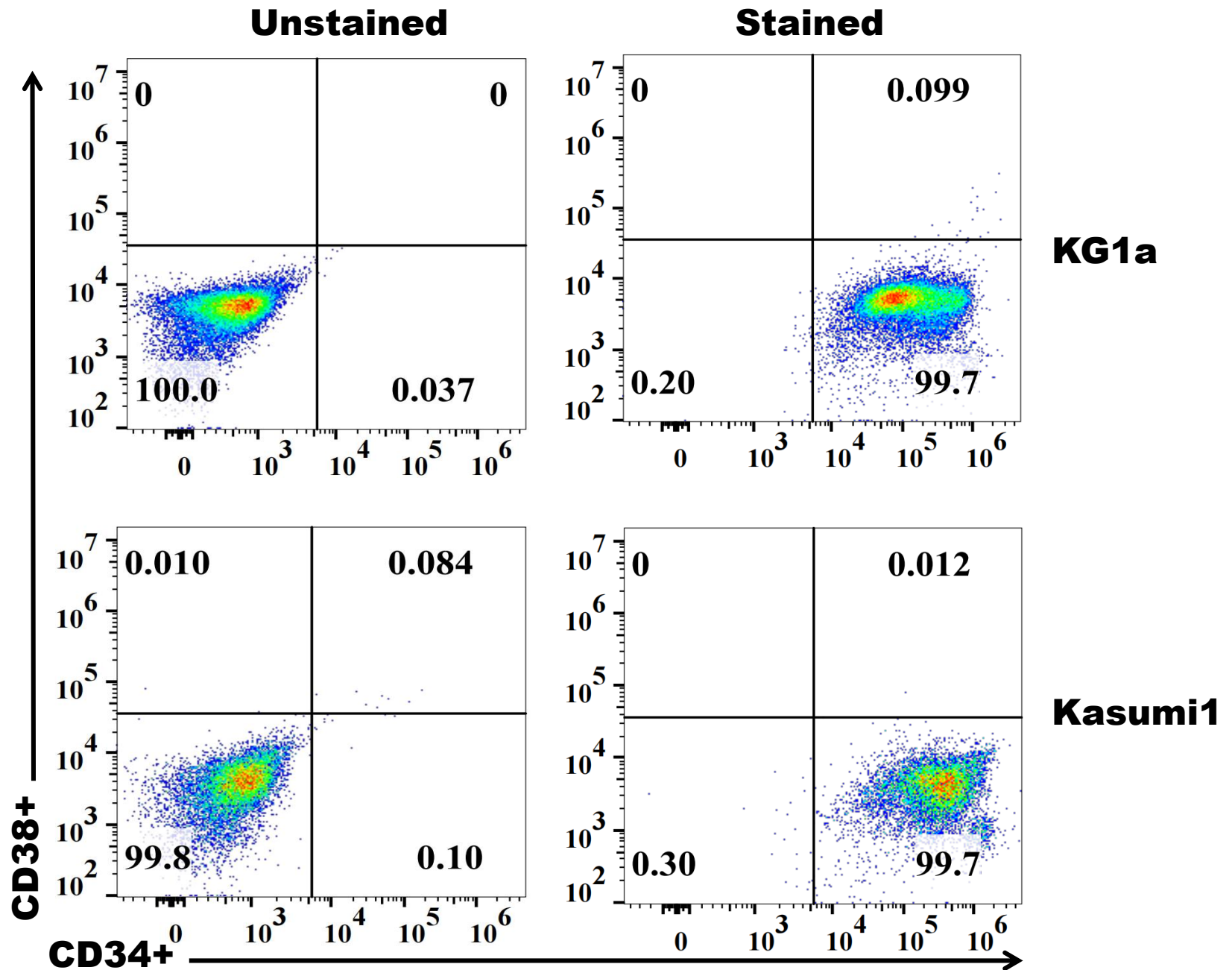

Supplement: Supplementary file 1 — Additional file 1: Supplementary Figure 1. The expression of CD34 and CD38 in KG1а and Kasumi-1 cells. [file 40164_2022_282_MOESM1_ESM.pdf]
